# Activation of Invariant NKT Cells Exacerbates Experimental Visceral Leishmaniasis

**DOI:** 10.1371/journal.ppat.1000028

**Published:** 2008-02-29

**Authors:** Amanda C. Stanley, Yonghong Zhou, Fiona H. Amante, Louise M. Randall, Ashraful Haque, Daniel G. Pellicci, Geoff R. Hill, Mark J. Smyth, Dale I. Godfrey, Christian R. Engwerda

**Affiliations:** 1 Immunology and Infection Laboratory, Queensland Institute of Medical Research, Herston, Queensland, Australia; 2 Department of Microbiology and Immunology, University of Melbourne, Parkville, Victoria, Australia; 3 Cancer Immunology Program, Trescowthick Laboratories, Peter MacCallum Cancer Centre, East Melbourne, Victoria, Australia; Imperial College London, United Kingdom

## Abstract

We report that natural killer T (NKT) cells play only a minor physiological role in protection from *Leishmania donovani* infection in C57BL/6 mice. Furthermore, attempts at therapeutic activation of invariant NKT (iNKT) cells with α-galactosylceramide (α-GalCer) during *L. donovani* infection exacerbated, rather than ameliorated, experimental visceral leishmaniasis. The inability of α-GalCer to promote anti-parasitic immunity did not result from inefficient antigen presentation caused by infection because α-GalCer–loaded bone marrow–derived dendritic cells were also unable to improve disease resolution. The immune-dampening affect of α-GalCer correlated with a bias towards increased IL-4 production by iNKT cells following α-GalCer stimulation in infected mice compared to naïve controls. However, studies in IL-4–deficient mice, and IL-4 neutralisation in cytokine-sufficient mice revealed that α-GalCer–induced IL-4 production during infection had only a minor role in impaired parasite control. Analysis of liver cell composition following α-GalCer stimulation during an established *L. donovani* infection revealed important differences, predominantly a decrease in IFNγ^+^ CD8^+^ T cells, compared with control-treated mice. Our data clearly illustrate the double-edged sword of NKT cell–based therapy, showing that in some circumstances, such as when sub-clinical or chronic infections exist, iNKT cell activation can have adverse outcomes.

## Author Summary

Natural killer T (NKT) cells are a unique subset of T cells that can produce large quantities of inflammatory cytokines very rapidly upon stimulation. They are known to be strongly stimulated by a molecule called α-galactosylceramide (α-GalCer) that is derived from a marine sponge, and in this way α-GalCer is hoped to provide effective immunotherapy for a wide range of diseases. We attempted to stimulate NKT cells with α-GalCer in mice infected with *Leishmania donovani*, a protozoan parasite that causes a chronic disease known as visceral leishmaniasis in humans. *L. donovani* characteristically causes an acute resolving infection in the liver where NKT cells are abundant. Therefore, we hypothesised that by stimulating these cells with α-GalCer we would improve the rate of hepatic disease resolution. However, while α-GalCer administered prior to infection had no effect on hepatic parasite burden, α-GalCer administered during an established infection exacerbated hepatic disease, associated with a decrease in IFNγ-producing CD8^+^ T cells. These results are important as they demonstrate that therapies aimed at modulating NKT cell function are not always beneficial, and adverse consequences may occur in certain circumstances, such as in the presence of persistent and/or sub-clinical infections.

## Introduction

Natural killer T (NKT) cells are a unique subset of CD1d-restricted T cells that provide a link between innate and adaptive immune responses. In mice, invariant NKT (iNKT) cells express a semi-invariant TCR, consisting of a Vα14Jα18 TCR α-chain and a TCR β-chain biased towards Vβ8.2, Vβ2, and Vβ7 expression (reviewed in [1]). Type II NKT cells are another cell subset in mice with more diverse TCR expression [1]–[3]. Upon stimulation, iNKT cells rapidly produce large quantities of pro- and anti-inflammatory cytokines, resulting in activation of other immune cells such as NK cells and conventional T cells [4]–[7] (also reviewed in [8]). NKT cells recognise and respond to glycolipid antigens presented on CD1d molecules. The most well-defined antigen for iNKT cells is α-galactosylceramide (α-GalCer), a marine sponge-derived glycolipid that specifically targets iNKT cells and no other lymphocyte populations directly [9]. The activation of iNKT cells by α-GalCer can enhance resistance in several infectious disease models, including viral, bacterial and parasitic infections (reviewed in [10]–[12]). Among parasitic infections studied, α-GalCer has been shown to enhance resistance to malaria [13], trypanosomiasis [14] and toxoplasmosis [15]. The ability of iNKT cells to produce IFNγ following stimulation with α-GalCer is important for this therapeutic effect and host protection during infection, although the robust induction of TNF, IL-4 and IL-13 by iNKT cells also occurs (reviewed in [8],[16],[17]. However, the connection between therapeutically induced NKT cell responses and physiological NKT cell responses is not always clear. Nevertheless, there are parallels between physiological and therapeutic NKT cell responses in some disease models. For example, in experimental tumour models, the growth of methylcholanthrene (MCA)-induced sarcoma cell lines is restricted by physiological IFNγ produced by endogenous NKT cells [18], while many other experimental cell lines, including the B16-F10 melanoma are only controlled by NKT cells following therapeutic activation with α-GalCer, again in an IFNγ-dependent manner [7]. It is also possible that following pathogen challenge, NKT cells will be exposed to foreign glycolipids, or self glycolipids, that, in the inflammatory environment, can trigger NKT cell responses that are similar to those induced by α-GalCer [19]–[22] (reviewed in [23]).

Visceral leishmaniasis (VL) is a potentially fatal human disease caused by infection with *Leishmania donovani* or *L. infantum (chagasi)* [24],[25]. Infection of genetically susceptible mice with *L. donovani* results in an acute but ultimately resolving infection in the liver associated with the development of granulomas around infected Kupffer cells [26]. In contrast, a chronic infection develops in the spleen, associated with severe immunopathology [27],[28]. Parasite numbers increase more slowly in the spleen, and total splenic parasite burdens usually only reach 5–10% of maximum levels in the liver, often with greater variation between individual mice than in the livers of the same animals [28],[29]. NKT cells have been shown to regulate CXCL10 expression in the livers of C57BL/6 mice in the early stages of *L. donovani* infection, an event thought to be important for hepatic granuloma development [30]. A subset of mouse NKT cells has also been reported to be activated by *L. donovani* lipophosphoglycan presented by host CD1d [31]. Studies in CD1d-deficient BALB/c mice have also suggested that NKT cells are important for the efficient control of *L. donovani* growth [31]. In addition, infection of immature human DC with *L. infantum* results in increased CD1d cell surface expression, and subsequent increased recognition and killing by IFNγ-producing iNKT cells [32]. CD4^+^ NKT cells have also been implicated in protection against the early stages of cutaneous leishmaniasis caused by *L. major* infection in genetically resistant mouse strains [33]. However, a more recent study has shown that clinical recovery from *L. major* infection can occur in NKT cell-deficient mice in the same time period as wild type mice, despite this early NKT cell role in protection [34].

Here we investigated the role of NKT cells during experimental VL in C57BL/6 mice, and tested whether stimulation of iNKT cells with α-GalCer could enhance anti-parasitic activity. Our data indicate that NKT cells are neither required for the development of immunity in the liver, nor for the control of parasite burden in the spleen following *L. donovani* infection. Furthermore, iNKT cell activation by α-GalCer hinders disease resolution in the liver. These results have important implications for the modulation of iNKT cell function during established disease.

## Results

### The Role of NKT Cells in the Control of *L. donovani* Infection in C57BL/6 Mice

**Figure 1:**
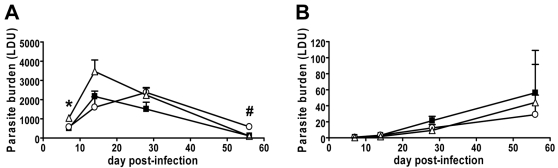
NKT Cells Are Not Required for Control of *L. donovani* Infection. Female C57BL/6 (closed squares), B6.Jα18^−/−^ (open circles) and B6.CD1d^−/−^ (open triangles) mice were infected with *L. donovani*, and parasite burdens were monitored from day 7 to day 56 p.i. in the liver (A) and spleen (B). Data represent the mean±SEM of parasite burdens (LDU) from four mice per group for each time point. One representative experiment of two performed with similar outcome is shown. Significant differences of *p*<0.05 between C57BL/6 and B6.Jα18^−/−^ mice (#) and C57BL/6 and B6.CD1d^−/−^ mice (*) are indicated.

To determine the relative roles of iNKT cells and type II NKT cells during experimental VL, we infected C57BL/6 mice deficient in iNKT cells only (B6.Jα18^−/−^) and all CD1d-restricted NKT cells (B6.CD1d^−/−^) with *L. donovani* and compared their course of infection to C57BL/6 controls. Only minor differences in parasite burdens were found between NKT cell-deficient mice and C57BL/6 controls. B6.Jα18^−/−^ mice had significantly higher hepatic parasite burdens than C57BL/6 mice at day 56 post-infection (p.i.) (*p*<0.05; Figure 1A), suggesting that iNKT cells may be required for optimal control of parasite growth at later stages of infection. However, C57BL/6 and B6.Jα18^−/−^ mice had resolved hepatic infection to a similar extent by day 90 and 180 p.i. (liver parasite burdens were 93±42 Leishman-Donovan units (LDU) versus 119±36 LDU in C57BL/6 versus B6.Jα18^−/−^ mice at day 90 p.i., and 0.4±0.5 LDU versus 6.2±3.0 LDU in C57BL/6 versus B6.Jα18^−/−^ mice at day 180 p.i.). A small but significant difference (*p*<0.05) in hepatic parasite burdens was also observed between B6.CD1d^−/−^ and C57BL/6 mice at day 7 p.i. (Figure 1A) suggesting a minor role for type II NKT cells in the early control of *L. donovani* growth. Alternatively, the CD1d molecule itself may signal to antigen presenting cells independently of NKT cells [35], thereby contributing to anti-parasitic immunity. B6.CD1d^−/−^ mice had resolved hepatic *L. donovani* infection to the same extent as C57BL/6 control mice by day 56 p.i. (Figure 1A), and were not examined after this time point. There was no decrease in the total numbers of IFNγ- or TNF-producing cells in the liver measured by ELISPOT at any time point in any group of mice (Figure S1). In addition, parasite burdens in the spleen were similar in all three groups of mice throughout the first 56 d of infection (Figure 1B) and until day 180 p.i. in C57BL/6 and B6.Jα18^−/−^ mice (splenic parasite burdens were 24±17 LDU versus 18±5 LDU in C57BL/6 versus B6.Jα18^−/−^ mice at day 90 p.i., and 5±3 LDU versus 10±3 LDU in C57BL/6 versus B6.Jα18^−/−^ mice at day 180 p.i.). Therefore, control of parasite growth in the liver was achieved in the absence of both iNKT and type II NKT cells, with only minor roles for these cells at different times following infection.

### Activation of iNKT Cells with α-GalCer at the Time of *L. donovani* Infection Does Not Improve Disease Resolution

**Figure 2:**
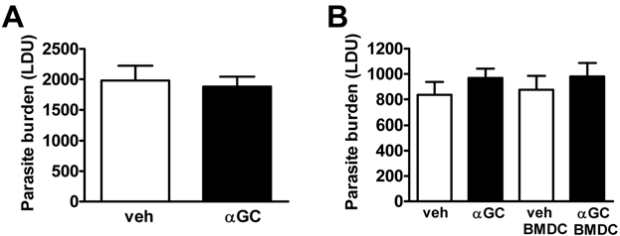
Stimulation of iNKT Cells with α-GalCer at the Time of Infection Does Not Enhance the Early Control of *L. donovani* Growth in the Liver. C57BL/6 mice were injected i.p. with either vehicle control (open bars) or 2 µg α-GalCer (closed bars) the day prior to *L. donovani* infection. Parasite burdens were determined at day 14 p.i. and data represent the mean±SEM of parasite burdens (LDU) from four mice per group (A). C57BL/6 mice were injected i.p. with either vehicle control (open bars) or 2 µg α-GalCer (closed bars), or i.v. with 5×10^5^ vehicle-pulsed BMDC (open bars) or α-GalCer-pulsed BMDC (closed bars) 2 h prior to *L. donovani* infection, as indicated (B). Parasite burdens were determined in the liver at day 14 p.i. and data represent the mean±SEM of parasite burdens (LDU) from four mice per group.

The links between physiological NKT cell responses and iNKT responses induced by ligands such as α-GalCer are not always apparent [8], and even though we were unable to detect a clear physiological role for NKT cells in protection against *L. donovani*, it was important to determine whether therapeutic iNKT cell activation with α-GalCer could improve disease resolution. Therefore, we next tested whether iNKT cells could be activated by α-GalCer at the time of infection to improve control of *L. donovani* growth. We focused these studies on the liver because NKT cells comprise a major cell population in this tissue site and parasite burdens are significantly higher and more consistent in the liver than in the spleen. The activity of α-GalCer was confirmed prior to all experiments by measuring IFNγ production by hepatic iNKT cells 2 h following α-GalCer administration (data not shown). A single injection of α-GalCer was administered so that the iNKT cell response was not tolerised or biased towards a Th2 response, as has previously been reported [12], [36]–[38]. First, α-GalCer was administered the day prior to infection, and no differences in hepatic parasite burdens were observed in treated mice, compared with controls on day 14 p.i. (Figure 2A). The same result was observed when α-GalCer was administered to mice 2 h prior to infection (Figure 2B). Stimulation with α-GalCer–loaded bone marrow–derived dendritic cells (BMDC) results in more prolonged cytokine responses than with soluble α-GalCer in mice due to selective targeting of the glycolipid to DC and improved presentation of the antigen to iNKT cells [37]. *L. donovani* has also been shown to interfere with antigen processing pathways [39]. Although there is no direct evidence for CD1d antigen presentation being compromised during experimental VL, we sought to eliminate this possibility by delivering α-GalCer–loaded BMDC to mice 2 h prior to infection. However, despite the stimulation of efficient iNKT cell activation by α-GalCer–loaded BMDC, demonstrated by increased serum cytokine levels (data not shown), and as previously reported [37] this mode of α-GalCer delivery also had no impact on hepatic parasite burdens relative to control mice that had received vehicle-loaded BMDC (Figure 2B).

### α-GalCer Treatment at the Time of iNKT Cell Activation in Response to *L. donovani* Infection Enhances Parasite Growth

**Figure 3:**
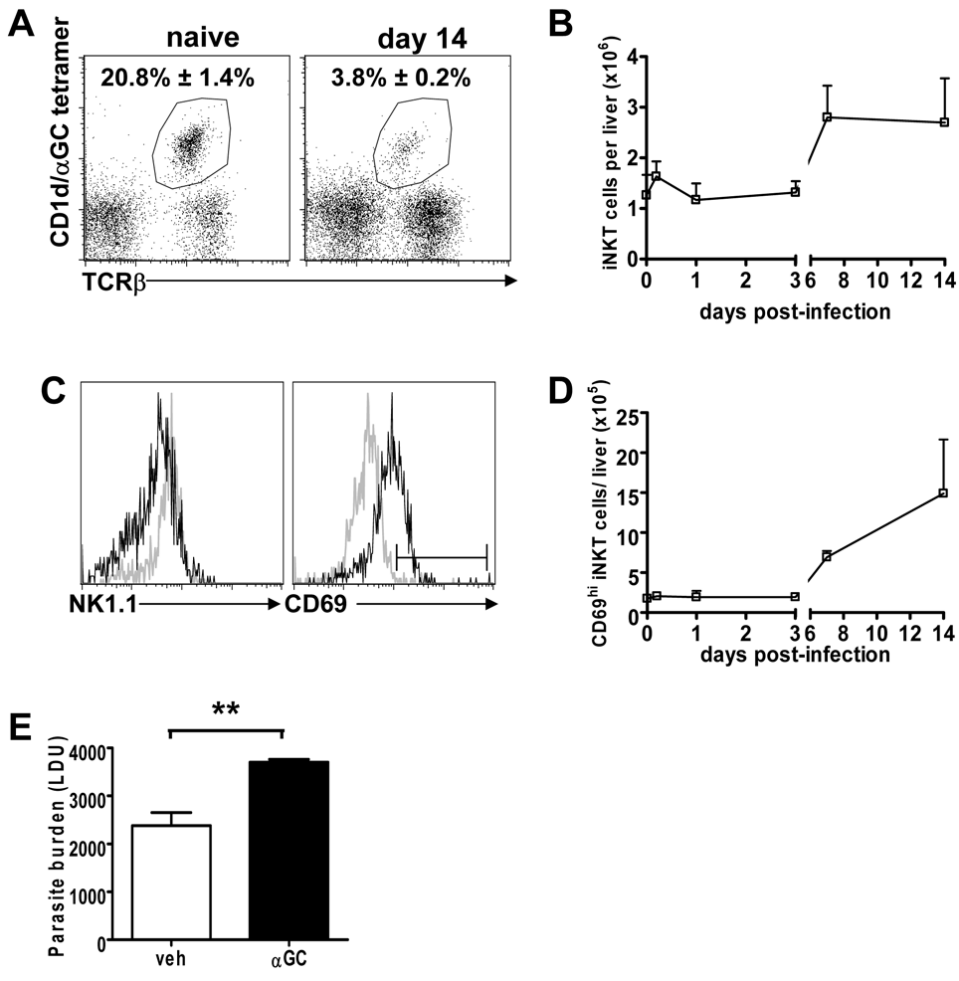
The Number of iNKT Cells and the Expression of CD69 by iNKT Cells Are Increased in the Liver by Day 7 after Infection, Yet Stimulation with α-GalCer at This Time Enhances Hepatic Parasite Growth. (A–D) C57BL/6 mice were infected with *L. donovani*, and killed at various time points p.i. for analysis of hepatic iNKT cells by flow cytometry. (A) iNKT cells were enumerated by labelling with CD1d/α-GalCer tetramers and anti-αβTCR, depicted for a naïve liver, and at day 14 p.i., as indicated. The numbers in the top right hand corner represent the mean±SEM of gated cells. (B) The absolute number of hepatic TCRβ^+^ tetramer^+^ iNKT cells at each time point. (C) The surface expression of NK1.1 and CD69 (early activation marker) is shown on gated hepatic NKT cells from a naïve animal (grey line) and at day 14 p.i. (solid black line). (D) Total number of hepatic NKT cells expressing enhanced levels of CD69, relative to naïve mice. All data are from three mice per time point, and data in (B) and (D) represent the mean frequency±SEM for each time point. (E) C57BL/6 mice were infected with *L. donovani* and treated with either vehicle control (open bars) or 2 µg α-GalCer (closed bars) on day 7 p.i. Parasite burdens were determined in the liver at day 14 p.i. and data represent the mean±SEM of parasite burdens (LDU) from four mice per group. Statistical differences of *p*<0.01 (**) for vehicle versus α-GalCer treatment are indicated.

We next determined whether and when iNKT cells were first activated in response to *L. donovani* infection, so that we could test whether α-GalCer stimulation at this time was effective at activating these cells for anti-leishmanial effects. Although the percentage of iNKT cells in the liver was reduced at day 14 p.i. (Figure 3A), due to the hepatic recruitment of other leukocytes during granuloma formation (reviewed in [26]), total iNKT cell numbers doubled by day 7 p.i., and this increase was sustained at day 14 p.i. (Figure 3B) due to an approximate 10-fold increase in total liver mononuclear cell (MNC) number. There was also evidence of TCR down-regulation by iNKT cells at day 7 p.i. (data not shown) and day 14 p.i. (Figure 3A), but not earlier, suggesting TCR-dependent NKT cell activation, as seen following α-GalCer stimulation [40]–[42]. Furthermore, NK1.1 expression on iNKT cells was also reduced at these times, consistent with activation of these cells (Figure 3C). The expression of CD69 on iNKT cells also increased above normal intermediate levels following *L. donovani* infection from day 7 p.i. onwards (Figure 3C and 3D). Together, these data suggested that day 7 p.i. was an appropriate time to stimulate iNKT cells with α-GalCer to coincide with the time when hepatic iNKT cells were naturally activated following *L. donovani* infection. However, when C57BL/6 mice were given α-GalCer at this time, there was a significant increase in hepatic parasite burdens on day 14 p.i. (*p*<0.05), relative to controls (Figure 3E). Thus, α-GalCer–stimulation of iNKT cells at the time when activation occurred naturally during infection suppressed, rather than improved, control of parasite growth in the liver.

### Treatment with α-GalCer during an Established *L. donovani* Infection Impairs Disease Resolution in the Liver

**Figure 4:**
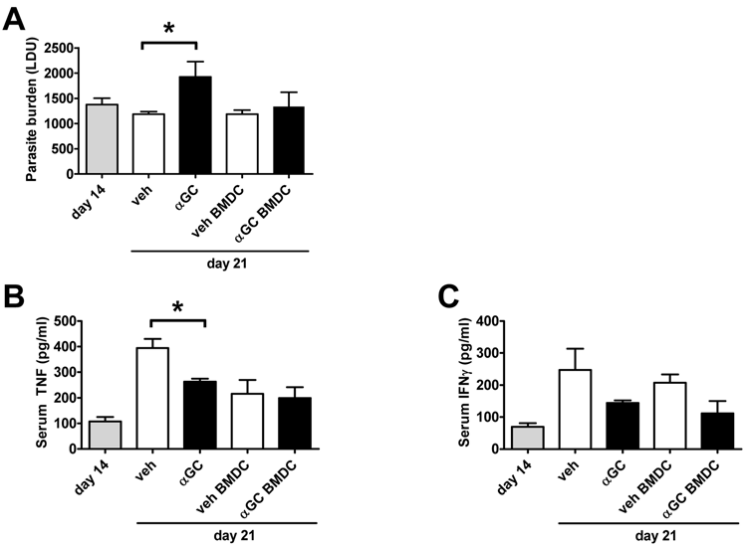
Stimulation of iNKT Cells with α-GalCer during an Established Infection Exacerbates *L. donovani* Growth in the Liver. C57BL/6 mice were infected with *L. donovani* and treated with either vehicle control (open bars) or 2 µg α-GalCer (closed bars) i.p., or injected i.v. with 5×10^5^ vehicle-pulsed BMDC (open bars) or α-GalCer-pulsed BMDC (closed bars) on day 14 p.i.. Parasite burdens were determined in the liver at day 14 p.i. in untreated mice (grey bars; base-line parasite burden) and 1 wk later in treated groups, as indicated (A). Data represent the mean±SEM of parasite burdens (LDU) from four mice per group. Serum TNF (B) and IFNγ (C) levels at indicated time points are also shown. Statistical differences of *p*<0.05 (*) for vehicle versus α-GalCer treatment are indicated.

To test whether the activation of iNKT cells during an established *L. donovani* infection had any therapeutic potential, α-GalCer was administered on day 14 p.i., which is around the time of peak hepatic parasite burden in C57BL/6 mice (Figure 1A). Mice were sacrificed 1 wk later, and again, there were significant increases (*p*<0.05) in hepatic parasite burdens in mice that received α-GalCer, compared to controls (Figure 4A). When α-GalCer–loaded BMDC were used instead of soluble α-GalCer, there were no significant changes in hepatic parasite burdens, compared with controls that received vehicle-loaded BMDC (Figure 4A). Serum TNF levels were reduced in α-GalCer-treated mice on day 21 p.i. compared to controls (*p*<0.05), but this did not occur in mice that received α-GalCer-loaded BMDC compared to controls that received vehicle-loaded BMDC (Figure 4B). A similar trend was observed for reductions in serum IFNγ levels in mice that received either soluble α-GalCer or α-GalCer–loaded BMDC compared to controls, but these differences were not statistically significant (Figure 4C). It should also be noted that vehicle-pulsed BMDC caused small reductions in serum TNF and IFNγ levels, relative to mice receiving vehicle alone (Figure 4B and 4C), suggesting that a DC-derived mediator might contribute to anti-protective effects seen following α-GalCer treatment. Thus, stimulation of iNKT cells with α-GalCer during an established infection, either in soluble form or loaded onto DC, did not improve disease control, and furthermore, soluble α-GalCer enhanced parasite growth in mice with an established infection.

To determine whether α-GalCer–treated mice recovered at later time points, α-GalCer was administered on day 14 p.i., and mice were sacrificed on both day 21 p.i. and day 70 p.i. (Figure S2). At day 70 p.i., hepatic parasite burdens had diminished significantly in mice receiving either vehicle or α-GalCer, and there was no significant difference between the groups. Therefore, the anti-protective effects of a single dose of α-GalCer during an established infection were transient, and anti-parasitic mechanisms were ultimately re-established in treated mice.

### IL-10 Does Not Impede the Function of iNKT Cells During *L. donovani* Infection

**Figure 5:**
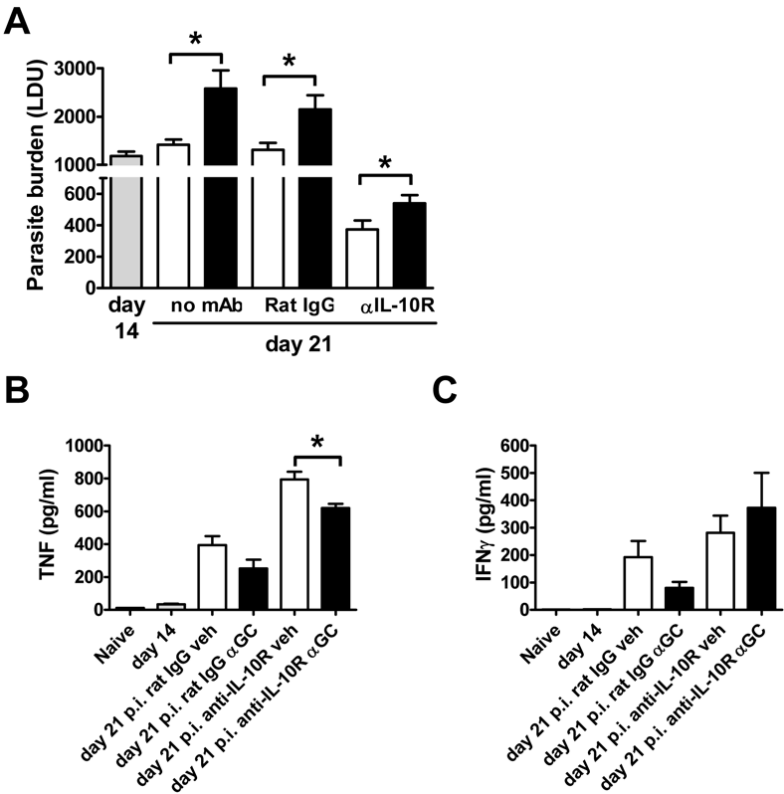
IL-10R Blockade Prior to α-GalCer Stimulation Does Not Improve Control of Parasite Growth During an Established Infection. (A) C57BL/6 mice were infected with *L. donovani* and treated with anti–IL-10R mAb or control rat IgG on day 12 p.i., as indicated. Two days later, mice were treated with vehicle control (open bars) or 2 µg α-GalCer (closed bars) i.p., and parasite burdens were determined in the liver 1 wk later. Parasite burdens were also determined in untreated mice at day 14 p.i. (grey bars; base-line parasite burden), and at day 21 p.i. in mice that received no antibody treatment, as indicated. Data represent the mean±SEM of parasite burdens (LDU) from four mice per group. (B) Serum TNF and (C) IFNγ levels at indicated time points are also shown. Statistical differences of *p*<0.05 (*) for vehicle versus α-GalCer treatment are indicated.

*L. donovani* infection results in the generation of immune regulatory mechanisms that prevent efficient disease resolution. In particular, IL-10 suppresses pro-inflammatory responses and parasite clearance during experimental VL [27], [43]–[45]. Previous work has reported an expansion of IL-10–producing CD4^+^ T cells in mice following *L. donovani* infection [46], and more recently, high levels of IL-10 mRNA accumulation were found in T cells isolated from splenic aspirates taken from Indian VL patients [47]. Furthermore, activated NKT cells are capable of IL-10 production [48]. Therefore, mice were treated with either anti–IL-10 receptor (IL-10R) mAb or control rat IgG on day 12 p.i., 2 d prior to the administration of α-GalCer. Liver parasite burdens were measured 1 wk following α-GalCer treatment. Again, the administration of α-GalCer to control mice on day 14 p.i. resulted in increased hepatic (*p*<0.05) parasite burdens compared to vehicle-treated controls (Figure 5A). IL-10R blockade was extremely potent in reducing parasite burdens (Figure 5A), as previously reported [43]. Stimulation of iNKT cells with α-GalCer in the absence of IL-10 signalling did not have an additive effect on the control of parasite growth, compared to vehicle-treated controls (Figure 5A), but instead resulted in a small, but significant (*p*<0.01), increase in hepatic parasite burdens (Figure 5A). IL-10 blockade led to significantly elevated levels of serum TNF in both vehicle and α-GalCer treated mice (*p*<0.01; Figure 5B), and a significant increase in serum IFNγ in mice that received α-GalCer (*p*<0.05; Figure 5C). However, α-GalCer treatment resulted in increased hepatic parasite burdens despite these increased levels of inflammatory cytokines. α-GalCer treatment reduced the levels of serum TNF but not IFNγ relative to vehicle treatment in mice that received IL-10R blockade. Thus, stimulation of iNKT cells with α-GalCer in the absence of IL-10 signalling during an established *L. donovani* infection did not reduce parasite burdens in the liver, thereby indicating that this mechanism of immune regulation was not responsible for the failure of α-GalCer to improve control of parasite growth.

### *L. donovani* Infection Alters iNKT Cell Cytokine and Hepatic Regulatory Cytokine Production in Response to α-GalCer Stimulation

**Figure 6:**
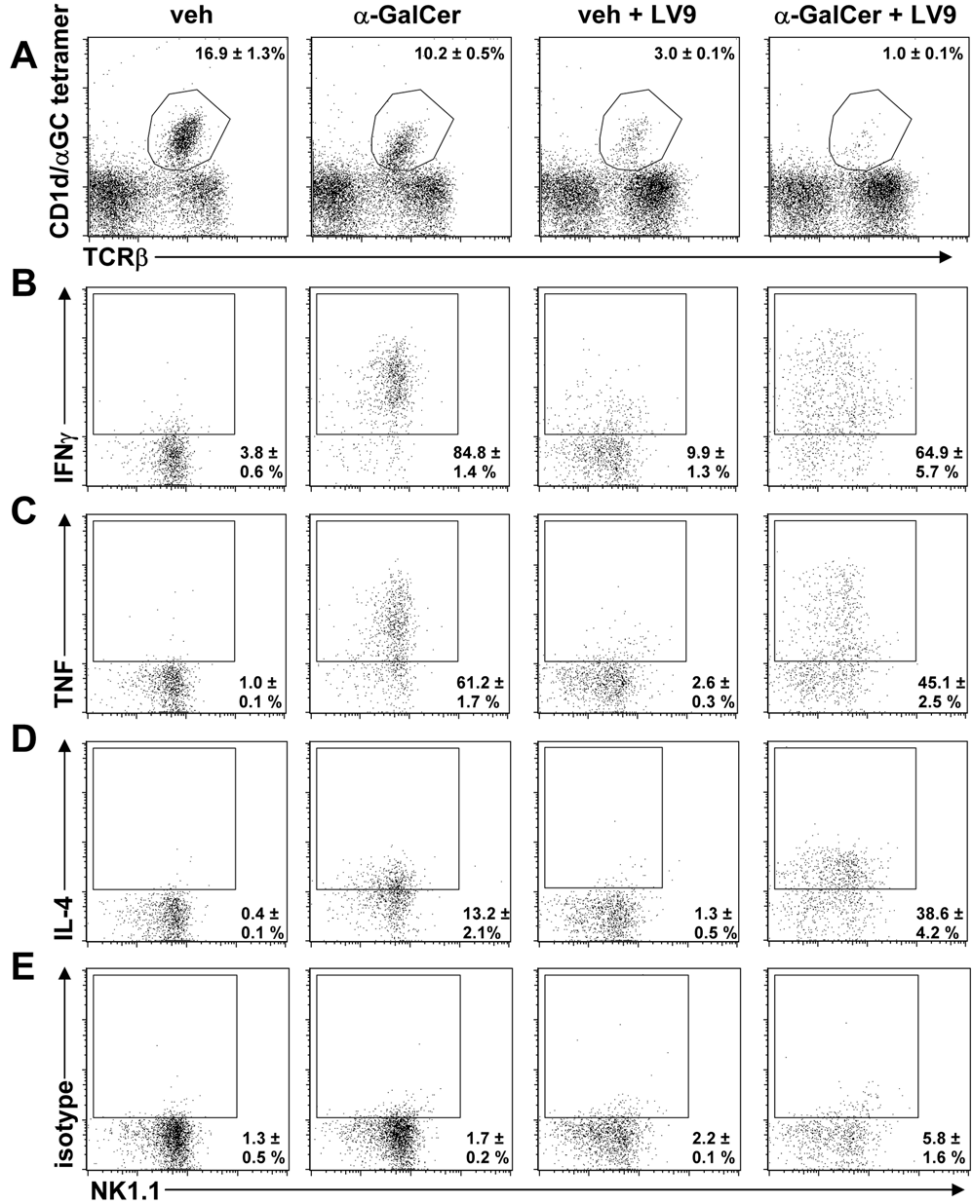
Stimulation of iNKT Cells with α-GalCer during an Established *L. donovani* Infection Results in a Decreased Frequency of Hepatic iNKT Cells Producing IFNγ and TNF, and an Increased Frequency of Hepatic iNKT Cells Producing IL-4. C57BL/6 mice were infected with *L. donovani* and on day 14 p.i., infected mice and uninfected controls were treated with vehicle control or 2 µg α-GalCer i.p. and killed 2 h later for FACS analysis of iNKT cell intracellular cytokine production. Hepatic lymphocytes were labelled with CD1d/α-GalCer tetramers and anti-αβTCR, and iNKT cells were electronically gated as shown (A). The mean±SEM of the percentage of gated cells for each group is shown in the top right-hand corner. From left to right data represent: naïve mice that received vehicle control, naïve mice that received α-GalCer, infected mice that received vehicle control on day 14 p.i., and infected mice that received α-GalCer on day 14 p.i.. Hepatic iNKT cells were examined for expression of NK1.1 and intracellular IFNγ (B), TNF (C), IL-4 (D), or rat IgG1 (E) (isotype control). Regions were set based on background levels of the isotype control as shown, and the numbers expressed in the bottom right hand corner are the mean±SEM in this region for each group (4 mice per group).

We next examined whether iNKT cells responded differently to α-GalCer stimulation during an established *L. donovani* infection. Naïve and infected mice (day 14 p.i.) were administered α-GalCer and sacrificed 2 h later. The down-regulation of TCR on iNKT cells following α-GalCer stimulation was noted in naïve mice (Figure 6A), as previously described [40], as was down-regulation of TCR on iNKT cells during experimental VL (Figure 6A). *L. donovani* infection did not alter the ratio of CD4^+^ to CD4^−^ iNKT cells in the liver, and therefore did not appear to selectively favour the expansion of a particular subset of iNKT cells (data not shown). IFNγ was readily detected in hepatic iNKT cells (Figure 6B), NK cells and conventional T cells (Figure S3) by day 14 p.i.. Following α-GalCer stimulation, the frequency of hepatic iNKT cells producing IFNγ and TNF was significantly reduced in infected animals relative to naïve animals (*p*<0.05), while the frequency of hepatic iNKT cells producing IL-4 was significantly increased (*p*<0.05; Figure 6B–6D). However, due to the increase in liver MNC number that had occurred by day 14 p.i. (3.3×10^6^±1.8×10^5^ versus 3.5×10^7^±7.9×10^6^ in naïve versus day 14 p.i., respectively), there was an approximate 3-fold increase in the absolute number of IL-4–producing hepatic iNKT cells, but little difference in the absolute numbers of IFNγ-producing and TNF-producing iNKT cells following α-GalCer stimulation in infected mice relative to α-GalCer–treated naïve controls. These data suggest an iNKT cell bias towards Th2 cytokine production in the liver following α-GalCer stimulation during *L. donovani* infection. Importantly, there was also an increase in the number of hepatic NK cells producing IFNγ following α-GalCer stimulation in infected animals relative to α-GalCer–treated naïve animals (*p*<0.05; 8.2×10^4^±5.3×10^3^ versus 6.7×10^5^±1.7×10^5^ in naïve versus day 14 p.i., respectively), indicating that *L. donovani* infection does not interfere with this important α-GalCer–mediated effector pathway.

**Figure 7:**
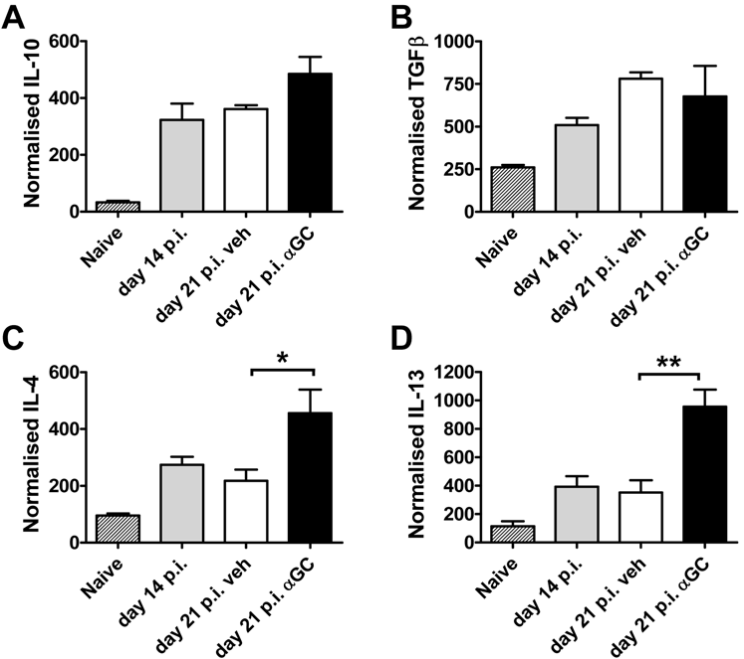
Th2 and Regulatory Cytokine mRNA Accumulation in the Liver Following α-GalCer Treatment during an Established *L. donovani* Infection. C57BL/6 mice were infected with *L. donovani* and treated with vehicle control (open bars) or 2 µg α-GalCer (closed bars) i.p. on day 14.p.i.. IL-10 (A), TGFβ (B), IL-4 (C), and IL-13 (D) mRNA accumulation were determined in the liver 1 wk later, as indicated. Cytokine mRNA accumulation was also determined in naïve mice (hatched bars) and untreated mice at day 14 p.i. (grey bars). Data represent the mean±SEM of cytokine mRNA accumulation from four mice per group. Statistical differences of *p*<0.05 (*) and *p*<0.01 (**) for vehicle versus α-GalCer treatment are indicated.

To determine whether there was a general enhancement in Th2 and/or regulatory cytokine production in response to α-GalCer treatment in *L. donovani*–infected mice, we next measured hepatic IL-10, TGFβ, IL-13 and IL-4 mRNA accumulation at day 21 p.i. in mice that had received either vehicle or α-GalCer at day 14 p.i. (Figure 7A–7D). We found no significant differences in hepatic IL-10 (Figure 7A) or TGFβ (Figure 7B) mRNA accumulation between vehicle- and α-GalCer–treated mice. Similar to the increased iNKT cell IL-4 production observed 2 h following α-GalCer administration shown above, we found a significant (*p*<0.05) increase in hepatic IL-4 mRNA accumulation (Figure 7C) 1 wk following α-GalCer administration, relative to vehicle-treated controls. Interestingly, we also found a similar increase (*p*<0.01) in hepatic IL-13 mRNA accumulation (Figure 7D) at the same time. Therefore, α-GalCer treatment of *L. donovani*–infected mice did not cause a general increase in Th2 and/or regulatory cytokine production, but instead, resulted in a selective increase in IL-4 and IL-13 production.

**Figure 8:**
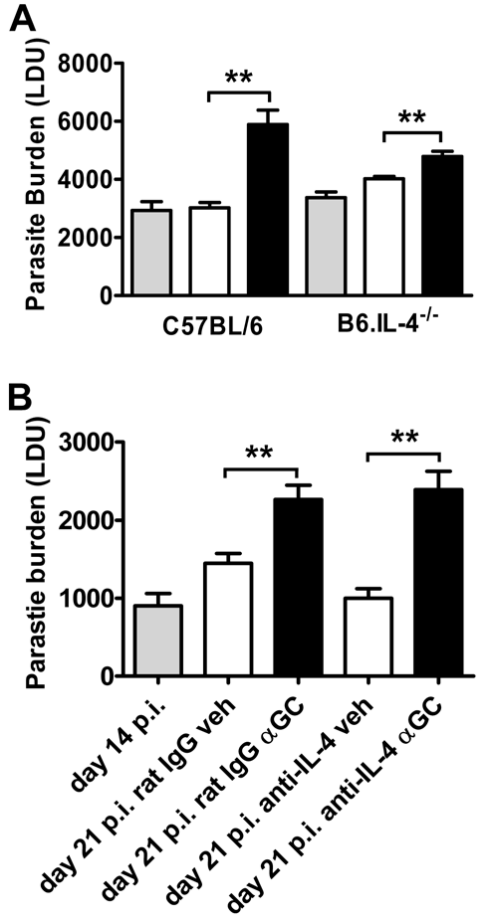
IL-4 Production following α-GalCer Stimulation during an Established *L. donovani* Infection Has Only a Minor Role in the Impaired Control of Parasite Growth. (A) C57BL/6 mice and B6.IL-4^−/−^ mice were infected with *L. donovani* and treated with vehicle control (open bars) or 2 µg α-GalCer (closed bars) i.p. on day 14 p.i., and parasite burdens were determined in the liver 1 wk later. Parasite burdens were also determined in untreated mice at day 14 p.i. (grey bars; baseline parasite burden). Data represent the mean±SEM of parasite burdens (LDU) from four mice per group. (B) C57BL/6 mice were also infected with *L. donovani* and treated with anti–IL-4 mAb or control rat IgG on days 14, 16 and 18 p.i., as indicated. Following mAb injection on day 14 p.i. mice were treated with vehicle control (open bars) or 2 µg α-GalCer (closed bars) i.p., and parasite burdens were determined in the liver 1 wk later. Parasite burdens were also determined in untreated mice at day 14 p.i. (grey bars; base-line parasite burden). Data represent the mean±SEM of parasite burdens (LDU) from four mice per group. Statistical differences of *p*<0.01 (**) for vehicle versus α-GalCer treatment are indicated.

We next tested whether increased IL-4 production following α-GalCer stimulation during an established *L. donovani* infection was responsible for impaired disease resolution. C57BL/6 and B6.IL-4^−/−^ mice were infected and administered α-GalCer or vehicle control 14 days later. At this time, B6.IL-4^−/−^ mice had similar hepatic parasite burdens to C57BL/6 mice (Figure 8A). α-GalCer treatment resulted in increased hepatic parasite burdens (*p*<0.05) in both C57BL/6 and B6.IL-4^−/−^ mice, compared to vehicle-treated controls (Figure 8A). To ensure that redundancies in IL-4 deficient mice were not influencing the outcome of these experiments, we also neutralised IL-4 activity in C57BL/6 mice using a mAb at a dose previously shown to improve control of *L. donovani* growth in a Th2 environment [49] (Figure 8B). Again, we found that *L. donovani*-infected mice treated with α-GalCer had significantly higher (*p*<0.01) hepatic parasite burdens, compared with vehicle controls, regardless of whether they received anti–IL-4 mAb or control rat IgG. Together, these data suggest that increased IL-4 production in response to α-GalCer treatment in *L. donovani*–infected mice, either by iNKT cells or another cell type following iNKT cell activation, could not alone explain the impaired clearance of parasites.

### Treatment with α-GalCer during an Established *L. donovani* Infection Alters Liver Cell Composition and Cytokine Production

**Figure 9:**
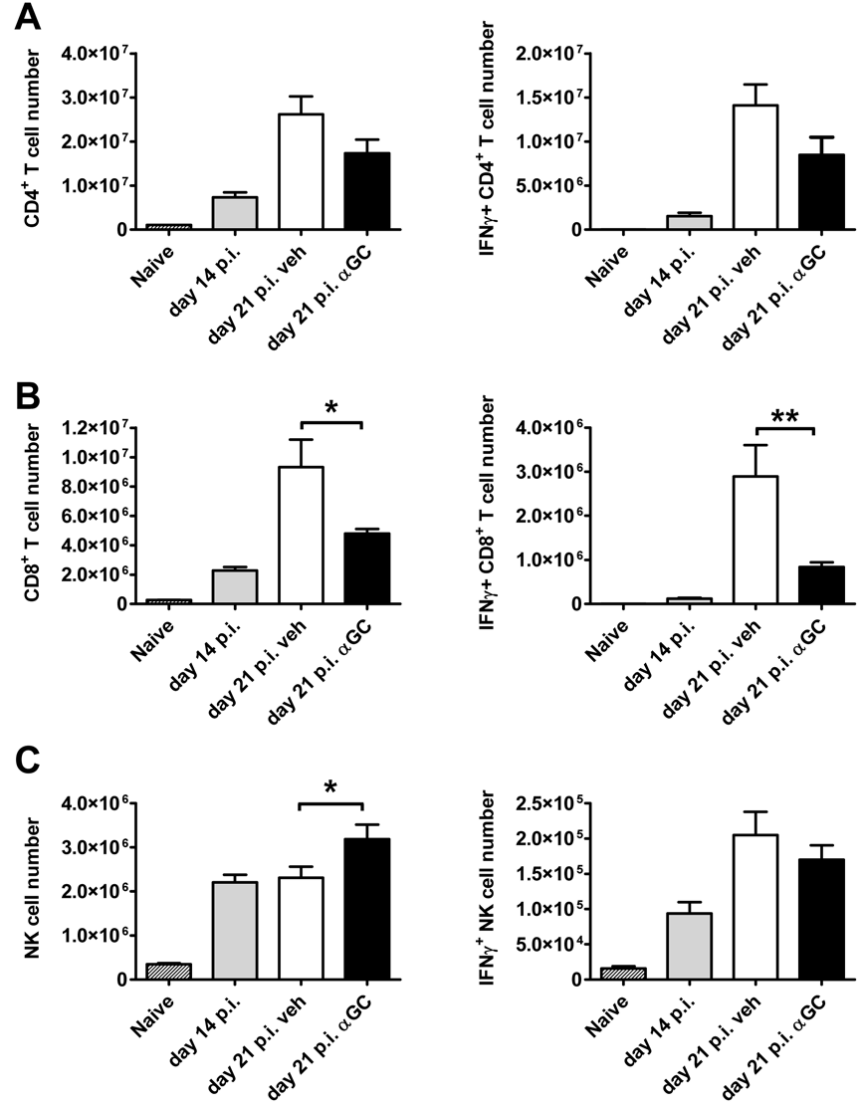
Changes in Liver Cell Composition and IFN_γ_^+^ Cell Numbers following α-GalCer Treatment during an Established *L. donovani* Infection. C57BL/6 mice were infected with *L. donovani* and treated with vehicle control (open bars) or 2 µg of α-GalCer (closed bars) i.p. on day 14 p.i. The number of hepatic CD4^+^ T cells (A), CD8^+^ T cells (B), and NK cells (C) were determined 7 d later, as was the number of these cells producing IFNγ, as indicated. Cell composition was also determined in naïve mice (hatched bars) and untreated mice at day 14 p.i. (grey bars). Data represent the mean±SEM of cell numbers from four mice per group. Statistical differences of *p*<0.05 (*) and *p*<0.01 (**) for vehicle versus α-GalCer treatment are indicated.

To test whether the activation of iNKT cells during an established *L. donovani* infection had any impact on the cellular composition of the liver that could explain the impaired parasite clearance, α-GalCer was administered on day 14 p.i., and the numbers of different hepatic mononuclear cell populations were assessed 7 d later. We found no significant differences in hepatic B cell, NKT cell, neutrophil, monocyte/macrophage or DC numbers in mice that had received α-GalCer, relative to vehicle-treated control animals (Figure S4). However, we did observe a small decrease in the number of hepatic CD4^+^ T cells (Figure 9A) and a larger, significant (*p*<0.05) decrease in the number of hepatic CD8^+^ T cells 7 d after α-GalCer-treatment (Figure 9B) relative to controls. We also found a small, but significant (*p*<0.05) increase in hepatic NK cell numbers at the same time (Figure 9C).

We next examined whether the activation status of various cell populations had been altered by α-GalCer administration at day 14 p.i.. We first focused on both hepatic DC and monocytes/macrophages and found no alterations in the expression of MHC-II, CD40, CD80 or CD86 on either population at 1, 2, 3, or 7 d following α-GalCer treatment, compared with vehicle-treated controls (data not shown). In addition, TNF production by monocytes/macrophages was not different between treatment groups at these times after infection, and no TNF was detected in DC (data not shown). Therefore, the activation status of the major hepatic antigen-presenting cell populations did not appear to be modulated by α-GalCer. To investigate whether lymphocyte activation had been altered, we examined IFNγ production following α-GalCer or vehicle treatment and found no difference in the number of hepatic IFNγ^+^ NK cells (Figure 9C) or IFNγ^+^ NKT cells (data not shown) by 7 d post-treatment. However, we did observe a small decrease in the number of IFNγ^+^ CD4^+^ T cells (Figure 9A) and a larger, significant (*p*<0.05) decrease in the number of IFNγ^+^ CD8^+^ T cells 7 d after α-GalCer-treatment (Figure 9B). Given the importance of IFNγ [50] and CD8^+^ T cells [51] for the effective resolution of hepatic *L. donovani* infection, this loss of IFNγ^+^ CD8^+^ T cells after α-GalCer treatment is a likely explanation for the enhanced parasite growth observed in our studies.

## Discussion

We report that CD1d-restricted NKT cells have only a minor physiological role in experimental VL in C57BL/6 mice. More importantly, our results indicate that intentional stimulation of iNKT cells with the glycolipid α-GalCer during *L. donovani* infection can exacerbate disease. This suggests that the therapeutic use of NKT cell activators, such as α-GalCer, may not always provide beneficial effects, particularly in situations where sub-clinical or chronic infection may be present.

Previous studies in CD1d-deficient BALB/c mice suggested that NKT cells were required for efficient control of hepatic *L. donovani* infection [31]. The most likely explanation for this discrepancy with our data is a different requirement for NKT cells in C57BL/6 and BALB/c mice, possibly reflecting different kinetics of parasite control between these mouse strains [28]. Alternatively, NKT cell responses may be influenced by host genetic background, as previously reported in experimental cerebral malaria [52]. Another study in B6.Jα18^−/−^ mice suggested that NKT cells were required for the efficient induction of CXCL10 production during the early stages of *L. donovani* infection in the liver [30]. However, given the limited differences observed in hepatic parasite burdens between B6.Jα18^−/−^ mice and control mice at early time points (Figure 1A), our data suggest that NKT-independent chemokine signals can compensate for this lack of early CXCL10 production.

Although only a limited physiological role was found for CD1d-restricted NKT cells during experimental VL, the activation of these cells has been shown to improve disease outcome in many infections, often via the increased production of IFNγ [11]–[15],[53]. This can occur even when there appears to be little requirement for NKT cells during natural infection, and is often due to downstream activation of NK cells. For example, although iNKT cells have only a minor role in the clearance of murine cytomegalovirus, the activation of iNKT cells by α-GalCer during infection resulted in reduced viral replication in visceral organs [53]. However, despite iNKT cell IFNγ and TNF production, and enhanced NK cell IFNγ production in the liver following α-GalCer administration during infection, the control of *L. donovani* was not improved. In fact, α-GalCer treatment during an established infection suppresses parasite clearance from the liver. In contrast, the administration of another strong inducer of a Th1 response, recombinant IL-12, does enhance disease resolution in experimental VL [54], indicating that α-GalCer has a different qualitative effect despite its ability to induce a strong Th1 response in *L. donovani*-infected mice. This may be related to the ability of α-GalCer to stimulate both Th1 and Th2 cytokine production.

The reason for the different effects of α-GalCer stimulation on parasite growth before and after infection is unclear, but it seems likely that changes in local tissue environments during infection may alter the outcome of iNKT cell activation with an impact on anti-leishmanial immunity. This explanation is supported by the finding that the timing of α-GalCer administration was also critical to the effect on murine experimental conjunctivitis [55]. The effect of other regimes of α-GalCer therapy, other sites of administration and/or other analogues such as α-C-GalCer [56],[57] have not yet been tested in experimental VL, and it remains possible that these other strategies might represent a therapeutic regime by which iNKT cell activation may improve disease resolution in this model. However, our data clearly show that α-GalCer therapy can exacerbate disease in *L. donovani*-infected mice.

A major functional change following α-GalCer-stimulation during *L. donovani* infection in the current study was rapidly increased numbers of IL-4 producing hepatic iNKT cells and a sustained increase in hepatic IL-4 mRNA accumulation 1 wk later. However, when *L. donovani*–infected B6.IL-4^−/−^ mice were treated with α-GalCer, hepatic parasite burdens were still increased compared to vehicle-treated B6.IL-4^−/−^ mice. A similar failure to reverse the immune-dampening effect of α-GalCer was also found when mice were treated with anti–IL-4 mAb before and after α-GalCer treatment. Therefore, the increased α-GalCer induced IL-4 production by iNKT cells or another hepatic cell population during *L. donovani* infection could not alone explain the enhanced hepatic parasite growth. It is possible that the induction of other Th2 and/or regulatory cytokines by α-GalCer might contribute to the increased parasite burden observed. Although no α-GalCer–related increase in hepatic IL-10 or TGFβ mRNA accumulation was found, we did observe a significant increase in IL-13 mRNA accumulation. Studies in IL-13–deficient mice indicate that IL-13 itself does not influence resistance to *L. donovani* infection [58]. However, the enhanced production of both IL-4 and IL-13 may work together to exacerbate parasite growth. This possibility is currently being examined.

Other major changes observed in α-GalCer–treated infected mice were alterations to liver cell composition. Total hepatic NK cell number increased in mice that received α-GalCer, yet the number of IFNγ^+^ NK cells remained similar to vehicle-treated mice. NK cells can produce regulatory cytokines and modulate antigen presenting cell function (reviewed in [59]) and in this way NK cells may have contributed to the enhanced parasite growth seen following α-GalCer treatment. Studies to further elucidate the impact of NK cells on the immune dampening effect of α-GalCer in *L. donovani*-infected mice are underway. The other significant change in liver cell composition that occurred in α-GalCer–treated mice was a decrease in the number of CD8^+^ T cells, and importantly, a decrease in the number of IFNγ^+^ CD8^+^ T cells. Previous studies by Murray and colleagues have demonstrated a critical role for both CD8^+^ T cells [51] and IFNγ [50] for the effective resolution of hepatic *L. donovani* infection. Thus, it is likely that such a change to CD8^+^ T cell effector numbers will have a major impact on the ability of an animal to control parasite growth. The reason for this reduction in CD8^+^ T cell number is currently unknown, but could involve enhanced activation-induced cell death (AICD) and/or failure of expansion in this cell population. These possibilities are being investigated.

In summary, our data indicate that although NKT cells have a relatively minor physiological role in experimental VL, intentional stimulation of iNKT cells with α-GalCer can exacerbate infection. This finding is significant because it highlights the fact that therapies aimed at modulating NKT cell function are not always beneficial to the host, and it is important to take into account the possibility of adverse consequences, such as may occur in the presence of pre-existing infections.

## Materials and Methods

### Mice

Female C57BL/6 and BALB/c mice were purchased from the Australian Resource Centre (Canning Vale, Western Australia), and maintained under conventional conditions. B6.RAG1/J^−/−^ mice were bred at the Queensland Institute of Medical Research. B6.Jα18^−/−^ mice [60] were bred at the Peter MacCallum Cancer Centre and the Queensland Institute of Medical Research. B6.CD1d^−/−^ mice [61] and B6.IL-4^−/−^ mice (B6 Il4^tm1Nnt^, The Jackson Laboratory, Bar Harbor, ME) [62] were bred at the Peter MacCallum Cancer Centre. These mouse strains were originally generated on either the C57BL/6 background or the 129sv background and backcrossed 10 or more generations to C57BL/6 mice before use. Mice used were sex- and age-matched (6–10 wk), and all animal procedures were approved by the Queensland Institute of Medical Research Animal Ethics Committee.

### Parasites and infection of mice

*L. donovani* (LV9) was maintained by passage in BALB/c or B6.RAG1/J^−/−^ mice, and amastigotes were isolated from the spleens of chronically infected mice. Mice were infected by injecting 2×10^7^ amastigotes i.v. via the lateral tail vein, killed at the times indicated in the text by CO_2_ asphyxiation and bled via cardiac puncture. Spleens and perfused livers were removed, and parasite burdens were determined from Diff-Quick-stained impression smears (Lab Aids, Narrabeen, Australia) and expressed in Leishman-Donovan units (LDU) (the number of amastigotes per 1,000 host nuclei multiplied by the organ weight in grams) [63]. Hepatic mononuclear cells (MNC) and splenocytes were isolated immediately following death as previously described [64],[65].

### Antibodies

Allophycocyanin (APC)-conjugated anti-TCRβ chain, phycoerythrin (PE)-conjugated anti-CD8α, PE-Cy5-conjugated anti-CD4, PE-conjugated anti-Ly6G, fluorescein isothiocyanate (FITC)-conjugated anti-Ly6C, APC-conjugated anti-B220, FITC-conjugated anti-CD19, APC-conjugated anti-CD11c, FITC-conjugated anti–I-A/I-E (MHC-II), PE-Cy5-conjugated anti-CD11b, PE-conjugated anti-CD69, PE-conjugated anti-IFNγ, PE-conjugated anti-TNF, PE-conjugated anti–IL-4, PE-conjugated rat IgG1, PE-conjugated rat IgG2a, and biotinylated anti-NK1.1 mAbs were purchased from Biolegend (San Diego, CA) or BD Biosciences (Franklin Lakes, NJ). PE-Cy5-conjugated α-GalCer–loaded or vehicle-loaded mouse CD1d tetramers were produced as previously described [66]. Biotinylated antibodies were detected using Alexa Fluor 488–conjugated streptavidin (Invitrogen Life Technologies, Eugene, OR). Anti–IL-10R (1B1.3a; rat IgG1) was purchased from BD Biosciences. Purified control rat IgG was purchased from Sigma-Aldrich (Castle Hill, Australia).

### Flow cytometry

The labelling of hepatic MNC for cell surface antigens and intracellular cytokines for FACS analysis was performed as previously described [40],[65]. Flow cytometric analysis was performed on a FACScalibur flow cytometer and analysed using Cell Quest Pro (BD Biosciences) and FlowJo Software (Tree Star, Ashland, OR). Cell populations in the liver were defined as follows: CD4^+^ T cells (CD4^+^ TCRβ^+^), CD8^+^ T cells (CD8α^+^ TCRβ^+^), B cells (B220^+^ CD19^+^), neutrophils (CD11b^+^ Ly6G^+^), macrophages/monocytes (CD11b^+^ Ly6C^+^), dendritic cells (DC; CD11c^hi^ MHCII^hi^), NK cells (NK1.1^+^ TCRβ^−^), NKT cells (CD1d α-GalCer tetramer^+^ TCRβ^+^ or NK1.1^+^ TCRβ^+^, as indicated in the text).

### In vivo α-GalCer treatment

α-GalCer (KRN7000) was provided by the Kirin Pharmaceutical Research laboratories (Kirin Brewery, Gumna, Japan) and was prepared in saline/0.5% (w/v) polysorbate-20. No endotoxin contamination was detected in the supplied material (Tomoaki Kuwaki, Kirin Brewery, personal communication). Mice received a single i.p. injection of 2 µg α-GalCer or vehicle control (saline/0.5% (w/v) polysorbate-20) at the times indicated in the text.

### Delivery of α-GalCer in bone marrow–derived dendritic cells (BMDC)

Bone marrow cells were isolated from the femurs of C57BL/6 mice and cultured in 20 ng/ml recombinant mouse GM-CSF and 20 ng/ml recombinant mouse IL-4 (R&D Systems, Minneapolis, MN), as previously described [37]. BMDC were supplemented with fresh media and 100 ng/ml α-GalCer or vehicle control on days 6–8 of culture. BMDC (5×10^5^) were injected i.v. at the times indicated in the text.

### IL-10 and IL-4 neutralisation

Mice were injected i.p. with 1 mg of anti-IL-10R mAb (1B1.3a) or purified control rat IgG (Sigma-Aldrich) at day 12 p.i.. Mice were injected i.p. with 1 mg of anti–IL-4 mAb (11B11) or purified control rat IgG on days 14, 16 and 18 p.i..

### Serum cytokine analysis

Serum samples were analysed for IFNγ and TNF using a cytometric bead array (mouse inflammation kit, BD Biosciences) on a FACScan cytometer equipped with Cell Quest Pro and CBA Software (BD Biosciences).

### ELISPOT analysis

The frequency of liver MNC producing IFNγ or TNF was determined by direct *ex vivo* ELISPOT assay, as previously described [67] using mAbs R46A2 and TN3-19.12, respectively, as capture antibodies and polyclonal anti-murine IFNγ or TNF as detecting antibodies (BD Biosciences).

### Real-time reverse transcriptase–polymerase chain reaction

Total RNA was extracted from liver tissue using TRIzol reagent (Invitrogen Life Technologies), and an RNeasy Mini Kit with on-column DNase digestion (Qiagen, Valencia, CA). RNA samples were reverse transcribed into cDNA using the High Capacity cDNA Reverse Transcription Kit (Applied Biosystems, Foster City, CA) according to the manufacturer's instructions. The number of IL-10, TGFβ, IL-4, IL-13 and HPRT (house-keeping gene) cDNA molecules in each sample were calculated using Taqman Gene Expression Assays (Applied Biosystems). All real-time RT-PCR were performed on a Corbett Research RG-3000 Rotor Gene (Corbett Life Sciences, Sydney, Australia). Standard curves were generated with known amounts of cDNA for each gene, and the number of cytokine molecules per 1,000 HPRT molecules in each sample was calculated.

### Statistical analysis

Statistical significance of differences in parasite burdens over a time course of infection were determined using a one way analysis of variance (ANOVA) followed by a Tukey's Post-Test. Statistical significance of other parameters were determined using a Mann-Whitney U test or Student's t-test. All analysis was conducted using GraphPad Prism version 4.03 for Windows (GraphPad Software, San Diego, CA) and *p*<0.05 was considered statistically significant.

## Supporting Information

Figure S1 The Number of IFNγ- or TNF-Producing Cells Is Similar in *L. donovani*-Infected C57BL/6 Mice and Mice Lacking NKT Cells. Female C57BL/6 (closed squares), B6.Jα18^−/−^ (open circles) and B6.CD1d^−/−^ (open triangles) mice were infected with *L. donovani*, and the total numbers of IFNγ- (A) or TNF-producing cells (B) in the liver were measured by ELISPOT from day 7 to day 56 p.i.. Data represent the mean ± SEM of cytokine-producing cells from four mice per group for each time point.
(1.24 MB TIF)

Figure S2 α-GalCer-Treated Mice Recover at Later Time Points. C57BL/6 mice were infected with *L. donovani* and treated with either vehicle control (open bars) or 2 µg α-GalCer (closed bars) i.p. on day 14 p.i.. Parasite burdens were determined in the liver at day 14 p.i. in untreated mice (grey bars; baseline parasite burden), 1 wk later in treated groups or 56 d later in treated groups, as indicated. Statistical differences of *p*<0.001 (***) for vehicle versus α-GalCer treatment are indicated (*n* = 4 mice per group).
(0.90 MB TIF)

Figure S3 IFNγ Is Readily Detected in Hepatic NK Cells and Conventional T Cells 14 Days After *L. donovani *Infection. Naïve C57BL/6 mice or mice infected with *L. donovani* were treated with vehicle control on day 14 p.i. and killed 2 h later for FACS analysis of intracellular IFNγ production, as indicated. Hepatic lymphocytes were labelled with CD1d/α-GalCer tetramers, anti-αβTCR, anti-NK1.1, and anti-IFNγ. T cells (A) and NK cells (B) were electronically gated as shown, and examined for expression of IFNγ or isotype control antibody. The percentage of gated cells is shown in the top left-hand corner. One representative animal of four examined is shown.
(5.38 MB TIF)

Figure S4 Liver Cell Composition Following α-GalCer Treatment. C57BL/6 mice were infected with *L. donovani* and treated with either vehicle control (open bars) or 2 µg α-GalCer (closed bars) i.p. on day 14 p.i.. Liver cell numbers were determined by FACS in naïve mice (hatched bars), at day 14 p.i. in untreated mice (grey bars), and 1 wk later in treated groups, as indicated (*n* = 4 mice per group).
(4.14 MB TIF)
