# Supplementary figures and images for: Activation of Invariant NKT Cells Exacerbates Experimental Visceral Leishmaniasis
Source: PLoS Pathog. 2008 Feb 29;4(2):e1000028. doi: 10.1371/journal.ppat.1000028 (PMC2265425; doi:10.1371/journal.ppat.1000028)

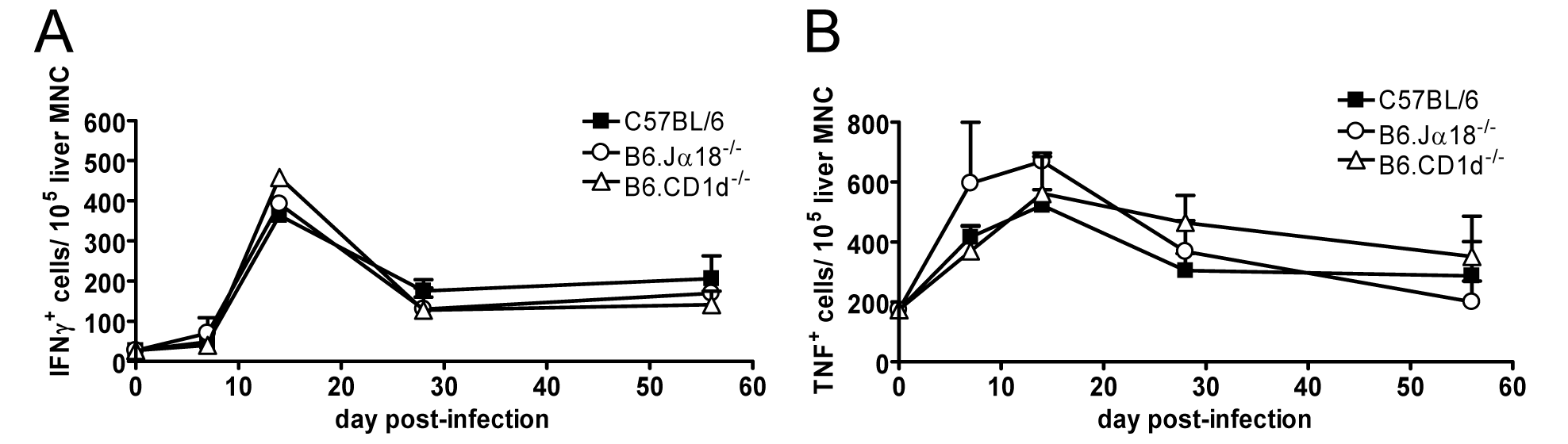

Supplement: Figure S1 — The Number of IFNγ- or TNF-Producing Cells Is Similar in L. donovani-Infected C57BL/6 Mice and Mice Lacking NKT Cells. Female C57BL/6 (closed squares), B6.Jα18−/− (open circles) and B6.CD1d−/− (open triangles) mice were infected with L. donovani, and the total numbers of IFNγ- (A) or TNF-producing cells (B) in the liver were measured by ELISPOT from day 7 to day 56 p.i.. Data represent the mean ± SEM of cytokine-producing cells from four mice per group for each time point. (1.24 MB TIF) [file ppat.1000028.s001.tif]

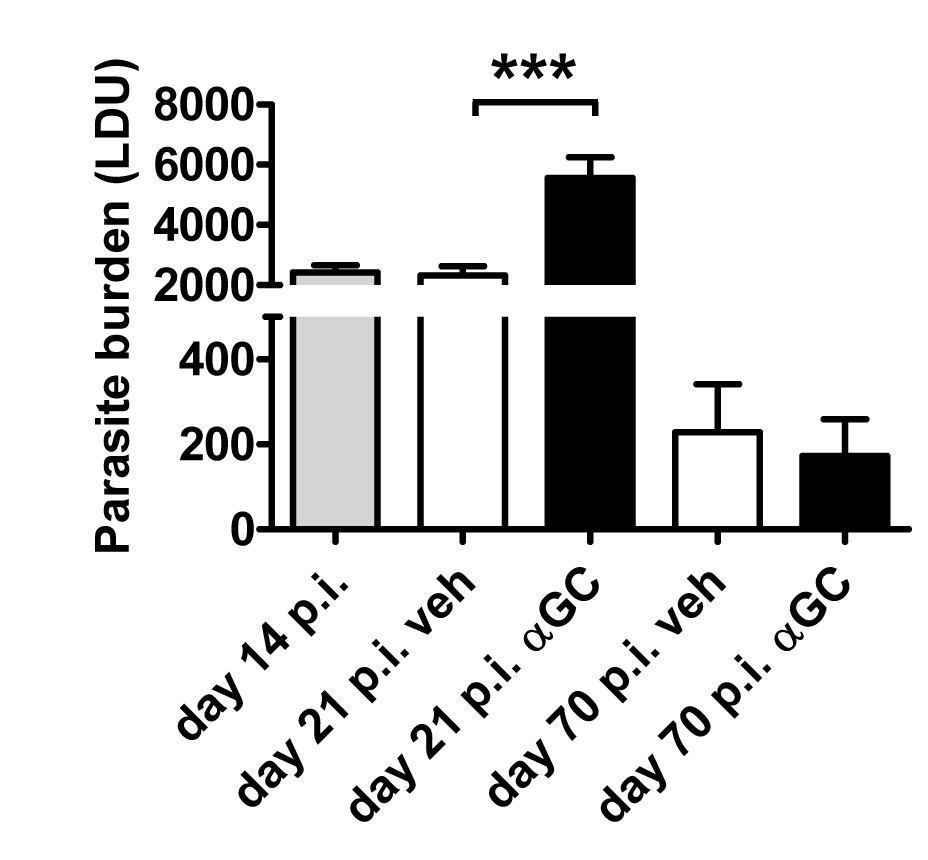

Supplement: Figure S2 — α-GalCer-Treated Mice Recover at Later Time Points. C57BL/6 mice were infected with L. donovani and treated with either vehicle control (open bars) or 2 µg α-GalCer (closed bars) i.p. on day 14 p.i.. Parasite burdens were determined in the liver at day 14 p.i. in untreated mice (grey bars; baseline parasite burden), 1 wk later in treated groups or 56 d later in treated groups, as indicated. Statistical differences of p<0.001 (***) for vehicle versus α-GalCer treatment are indicated (n = 4 mice per group). (0.90 MB TIF) [file ppat.1000028.s002.tif]

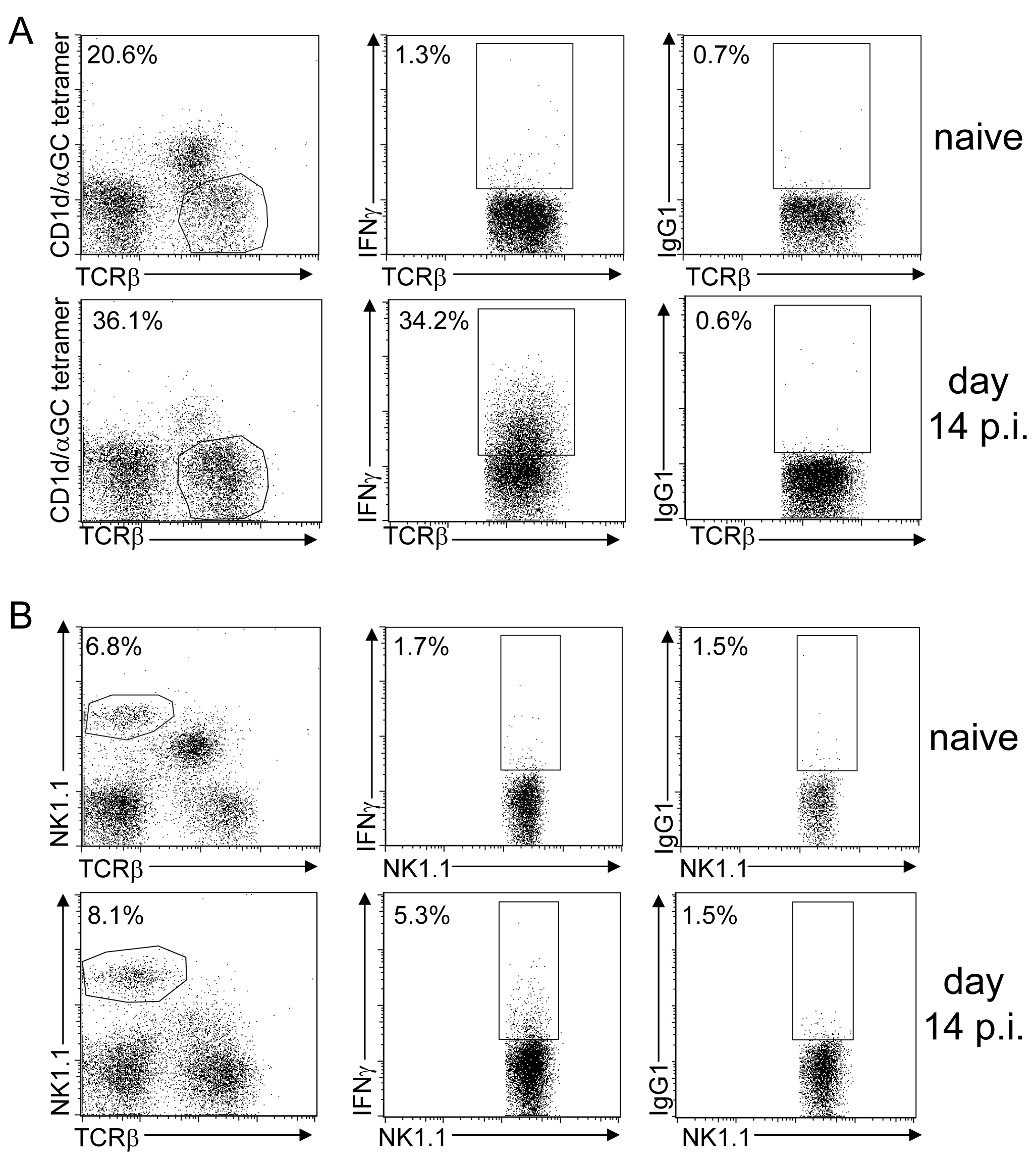

Supplement: Figure S3 — IFNγ Is Readily Detected in Hepatic NK Cells and Conventional T Cells 14 Days After L. donovani Infection. Naïve C57BL/6 mice or mice infected with L. donovani were treated with vehicle control on day 14 p.i. and killed 2 h later for FACS analysis of intracellular IFNγ production, as indicated. Hepatic lymphocytes were labelled with CD1d/α-GalCer tetramers, anti-αβTCR, anti-NK1.1, and anti-IFNγ. T cells (A) and NK cells (B) were electronically gated as shown, and examined for expression of IFNγ or isotype control antibody. The percentage of gated cells is shown in the top left-hand corner. One representative animal of four examined is shown. (5.38 MB TIF) [file ppat.1000028.s003.tif]

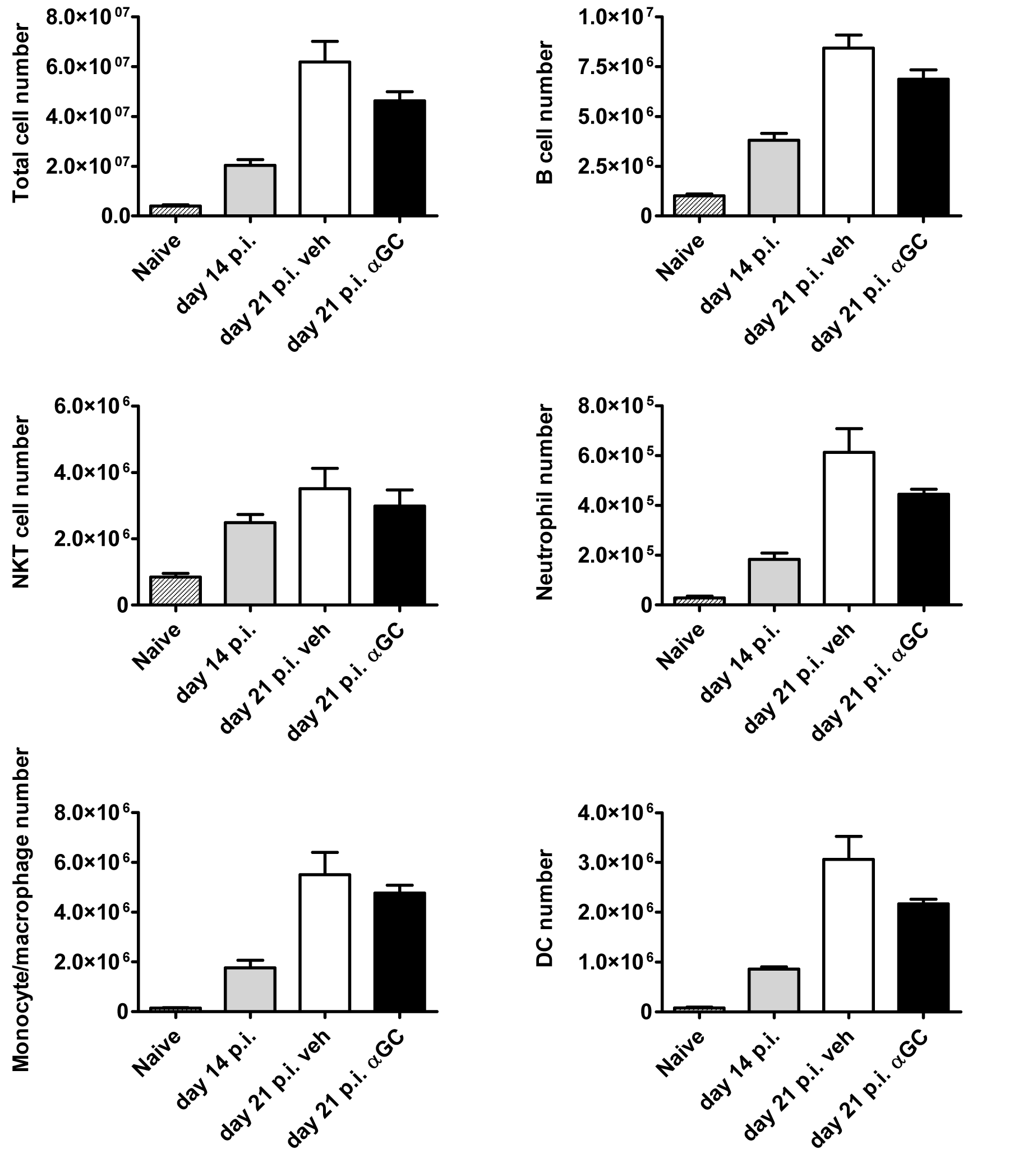

Supplement: Figure S4 — Liver Cell Composition Following α-GalCer Treatment. C57BL/6 mice were infected with L. donovani and treated with either vehicle control (open bars) or 2 µg α-GalCer (closed bars) i.p. on day 14 p.i.. Liver cell numbers were determined by FACS in naïve mice (hatched bars), at day 14 p.i. in untreated mice (grey bars), and 1 wk later in treated groups, as indicated (n = 4 mice per group). (4.14 MB TIF) [file ppat.1000028.s004.tif]
